# Implications for Care Relationships in Technology-Supported Stroke Rehabilitation: A Qualitative Interview Study

**DOI:** 10.1007/s11948-026-00625-9

**Published:** 2026-08-27

**Authors:** Julie Bechtel, Alena Buyx, Bettina M. Zimmermann

**Affiliations:** 1 Institute of History and Ethics in Medicine, TUM School of Medicine and Health, Technical University of Munich, Munich, Germany; 2 Institute for Biomedical Ethics, University of Basel, Basel, Switzerland; 3 Institute of History and Ethics in Medicine Munich, Ismaninger Str. 22, 81675 Munich, Germany

**Keywords:** Technology-supported rehabilitation, Health technologies, Qualitative research, Therapeutic relationship, Stroke, Physical therapy, Ethics of care, Care relationships, Embedded ethics

## Abstract

This study explores how technology-supported rehabilitation reshapes the relationships between patients and therapists. Additionally, it examines how empirical findings can inform early design decisions. Qualitative interviews with patients and therapists identified their perceptions of PhysioMio, a wearable sensor-based rehabilitation device that detects muscle activity to monitor rehabilitation progress and enable independent home-based training. Adapting an ethics of care perspective, we focus on the significance of therapeutic relationships when discussing ethical nuances that emerge with the introduction of technology. Our findings show that, while patients value enhanced opportunities for agency and involvement, they do not seek full detachment from therapists. The successful integration of technology into rehabilitation hinges on strong, trust-based relationships, in which therapists take on an expanded role as contextual evaluators. This illustrates that rather than replace physical therapists, technology demands them to assume new roles. While participants generally prioritized purposeful and beneficial data collection over abstract privacy concerns, this acceptance was challenged when monitoring risked shifting therapists’ roles toward judging supervisors. Visualizing the rehabilitation process through data raised fears of information overload and sparked mixed reactions regarding motivation and peer comparisons. These insights influenced design choices, including tailoring data granularity to specific purposes and reassessing whether comparative feedback supports or undermines motivation. In conclusion, the ethical use of technology to support recovery and strengthen care relationships must be embedded within trusting therapeutic relationships, and adapted attentively and responsibly to the individual’s context, needs, abilities and values.

## Introduction

Strokes are a leading cause of death and disability worldwide, and the number of stroke incidents is continually rising (Feigin et al., 2022). Upper limb dysfunction affects up to 75% of stroke survivors (Hubbard et al., 2015). The increasing demand for stroke rehabilitation services pushes the development of technology-supported rehabilitation devices (Ramanandi, 2020), hoping to bridge the resulting shortage in rehabilitation services and improve the delivery of healthcare (Mennella et al., 2024). Several studies of such technology-supported rehabilitation devices have been presented in existing literature. For example, Bu et al. (2022) developed a game-based device to support patients in regaining motor control of their upper limbs. A prototype designed by Yang et al. (2019) facilitates arm-reaching exercises through games and visual guidance. Nussbaum et al. (2019) conducted a systematic review of mobile health applications in rehabilitation, providing an extensive overview of available technologies. This paper assesses the ethical and social implications of another rehabilitation device called “PhysioMio”, a wearable sensor-based rehabilitation device that detects muscle activity to continuously monitor a stroke patient’s rehabilitation progress while allowing them to train independently at home (Ilg, 2025a, 2025b).

The introduction of technology into rehabilitation requires careful consideration of their ethical and social implications during the development, implementation, and use of these technologies, to maximize the potential of health technologies to improve health outcomes while avoiding the creation or reinforcement of inequalities (Floridi & Cowls, 2019; Rigby, 2019).

So far, several qualitative studies have investigated physical therapists’ and patients’ views on technology-supported rehabilitation. Elnady et al. (2018) found that both stroke survivors and therapists recognized the potential of wearable, robotic devices for the rehabilitation of the upper limbs. At the same time, they raised concerns about the devices’ comfort and practicality, emphasizing the need for them to be tailored to the individual’s level of impairment. Flynn et al. (2019) reported that, while professionals viewed technology-supported rehabilitation positively, they also highlighted barriers to implementation such as cost, space requirements, workflow disruption and the importance of adequate training. Laparidou et al. (2021) demonstrated that acceptance of rehabilitation robotics is influenced by performance expectancy, effort expectancy, and social influence, as well as factors such as enjoyment, supportive relationships with staff, personalization, and clear instructions. However, there has been less research into how technologies reshape physical therapy rehabilitation practices and relationships (Archibald & Barnard, 2018; Lucivero & Jongsma, 2018; Topol, 2019), or how users navigate and negotiate the ethical challenges that emerge. Even fewer studies explore both patients’ and healthcare professionals’ views (Jongsma et al., 2021). Our study addresses this gap by exploring the ethical and social implications of introducing PhysioMio from the perspective of stroke rehabilitation patients and physical therapists, and by examining how they respond to and manage these issues in practice. Moving beyond a focus solely on usability or acceptance, the research considers how digital tools can reconfigure the care environment, for instance by redistributing responsibilities, affecting autonomy and privacy, and shaping users’ self-perception.

Aiming to surface ethical nuances that might otherwise remain overlooked (Ramvi et al., 2023) we adopt ethics of care as our primary analytical lens. Originally developed in feminist moral philosophy, ethics of care offers a compelling addition to abstract, rule-based ethical frameworks by centering moral attention on the dynamics of human relationships, emotional responsiveness, and contextual sensitivity (Gilligan, 1982; Tronto, 1993). Rather than focusing on the sovereignty of each individual, ethics of care understands people as inherently embedded in webs of dependency within their care relationships. In this study, care relationships refer to the dynamic, interdependent relationship between patient and physical therapist, encompassing dimensions of trust, communication, power, emotional support, and the negotiation of autonomy and dependency. These dimensions are understood as inherently relational. They cannot be located in one actor alone, but emerge from the ongoing interaction between patient, therapist, and in this case, technology. Care must be situation-oriented and informed by individual needs (Maio, 2018). Care ethics is particularly well suited to examine how rehabilitation technologies like PhysioMio reshape the very personal and interdependent dynamics of care. Against this backdrop, this paper addresses the research question: Based on the case study of PhysioMio, how does technology-supported rehabilitation (re)shape care relationships between patients and physical therapists?

This consideration matters especially in start-up environments like PhysioMio, where formalized ethical procedures may be lacking and teams are small, fast-moving, and resource-constrained, making ethical challenges particularly complex (Knopf et al., 2023). There is still limited empirical research examining how ethical considerations are negotiated and integrated during the earliest stages of technological development, when foundational design decisions are being made (Stahl & Coeckelbergh, 2016).

Intending to ensure that the answers to these ethical questions proactively shape the ethical development of technology, the present study adopts an “embedded ethics” approach, integrating ethicists throughout the development process and fostering ethically critical thinking and collaboration with developers (McLennan et al., 2022). By conducting this study in parallel to the development of a PhysioMio prototype and collaborating with the developers of PhysioMio from an early development stage, our research aims to explore how ethical thinking can actively shape the technology in real time.

## Methods

### Study Context

This study took place alongside the early-stage development of PhysioMio, a wearable sensor-based rehabilitation device that detects muscle activity designed at the Institute of Micro Technology and Medical Device Technology (TUM). Following an embedded ethics approach (McLennan et al., 2022), this study was conducted during the early development stages of PhysioMio with the aim of integrating empirically informed ethical reflection early in the development process and bringing to light the perspectives of patients and physical therapists. The ethics commission of the Technical University of Munich approved this study (study no. 2023-418-S-KK). The COnsolidated criteria for REporting Qualitative research (COREQ) checklist was applied to report this study (Tong et al., 2007) and is attached in the supplementary material.

### Research Design (Setting, Recruitment, Data Collection)

We conducted a qualitative interview study (Stickley et al., 2022) with thirteen stroke patients and seven physical therapists who had experience of stroke rehabilitation. Stroke patients who were in a Bavarian rehabilitation center were informed about this study when participating in a separate clinical study to test the accuracy of an early sensor system for PhysioMio. Those who were interested in participating arranged an interview appointment at the rehabilitation facility. Interviewed patients had to have experienced a stroke. Their deficits ranged from very subtle fine motor skill deficits to hemiplegia. None of the patients had severe cognitive impairments. The physical therapists were recruited through personal networks and the German Association of Physical Therapy (*Deutscher Verband für Physiotherapie*, 2025) and had to have experience working in stroke rehabilitation in Germany. Beyond these inclusion criteria, no further demographic data such as age, gender, health history or professional background were systematically collected. Initially, three physical therapists agreed to participate in interviews. After a preliminary analysis and discussions within the research team, additional interviews were deemed necessary to explore whether additional themes emerged. Thus, four additional interviews were conducted using the same guide and procedures. As the additional interviews did not generate substantial new insights, data collection was concluded in January 2026, consistent with a pragmatic understanding of saturation (Low, 2019). As the participants only contacted us if they were interested in participating in the study, we do not know how many patients and physical therapists were presented with the study information and were not interested in participating.

Interviewees were informed that the study was being conducted as part of a doctoral dissertation and knew that the interviewer (the doctoral candidate, medical student, female) was in communication with the developers at PhysioMio, but that she was independent from them as a researcher. Participants knew that the goal of the study was to analyze their views on PhysioMio to shape the future development of the device. All potential participants received a study information leaflet and gave written consent to participate in this study. As of today, no participant has retracted their consent.

The interviews were conducted in German and based on two interview guides (one for patients, one for physical therapists, see supplementary material for translated interview guides). Interview guides contained open-ended questions about their perceptions and anticipated stances towards the PhysioMio device that allowed us to explore any relevant topics that arose during the interviews (Britten, 1995; Denny & Weckesser, 2022). As the interviews took place at such an early stage of development, a prototype of PhysioMio was not yet available. Instead, interviewees were shown pictures of the early sensor prototype and given verbal descriptions of the device. Interviews began with a broad invitation for participants to reflect on their prior experiences with, or general expectations of, technology-supported rehabilitation. They then focused on the care relationship between patients and therapists, and how it might be affected by such technology. Participants were asked to consider the potential use of the device at home, and we discussed their perceptions of artificial intelligence and data feedback, as well as the device’s potential role in providing active support. The participants met once with the interviewer, prior to the interview, no relationship was established with the participants. Transcripts were not returned to participants. They were, however, provided with an Email address they could use to contact the interviewer if they wanted to be informed once the study findings were published. All seven physical therapists used this opportunity; no patient did.

Interviews lasted between 20 and 100 min. They were conducted by JB between October 2023 and January 2026 at a Bavarian rehabilitation center, where patients were undergoing rehabilitation treatment at the time. Interviews with physical therapists took place online (five interviews) and at the interviewer’s workplace (two interviews). For all interviews, the interviewer and the interviewee were the only people present in the room. The interviews were audio-recorded and transcribed verbatim. The GDPR compliant and encrypted *f4 Audiotranskription* software was used to support transcription for a subset of interviews. All transcripts were then reviewed against the original recordings to correct errors and ensure accuracy. Field notes were taken after each interview, informing minor adaptations to the interview guide and interviewing approach where necessary. All identifying variables, including names and places, were removed prior to analysis (pseudonymization). Illustrative quotes were translated from German to English by the authors.

Findings from the interview study were shared proactively with the developers of PhysioMio during regular meetings to address ethically relevant aspects and to enact cooperation between engineers and ethicists. The study participants’ identities were not shared with the developers, ensuring confidentiality. In addition to these meetings, an interview with the developers of PhysioMio was conducted towards the end of the interview study to assess how our findings had influenced the development process (interview guide in supplementary material).

### Data Analysis and Reflexivity

All qualitative data were analyzed using a reflexive thematic analysis approach (Braun & Clarke, 2006; 2023). This iterative, non-linear process involved repeated engagement with the material through familiarization, coding and theme development. Inductive coding was conducted in Atlas.ti (version 8), allowing patterns to emerge from the data. Themes and content organization were discussed with the research team. Early readings revealed prominent themes concerning relational dimensions, interdependence, and autonomy. These recurring patterns led us to adopt the theoretical lens of ethics of care. Returning to the data through this lens enabled us to refine the emerging themes further and interpret them within a relational ethics framework. The evolving findings were regularly discussed within the interdisciplinary research team to ensure analytic depth and reflexivity. Ongoing findings were not shared with other interview participants. A simplified overview of the coding tree, illustrating the main themes and subthemes that emerged from the inductive analysis, is provided in the supplementary material.

One distinguishing feature of the thematic analysis approach by Braun and Clarke is the explicit recognition that the “researcher is subjective, situated, aware and questioning“ (Braun & Clarke, 2022). Following this approach, the researchers critically reflected on their influence throughout the study, making their subjectivity a resource, rather than a limitation (Gough & Madill, 2012; Luttrell, 2019). In this research project, one challenge related to researching an unfamiliar subject (Berger, 2013). The interviewer (JB) was a young, healthy researcher with no personal experience of stroke or rehabilitation, allowing her to approach the research with an emotional distance that enabled her to listen openly to diverse perspectives. However, this distance also meant that the interviewer sometimes lacked the experiential intuition to know when or how to probe deeper, particularly during patient interviews where subtle cues or hesitations might have required more nuanced prompting.

The interviewer’s same institutional affiliation as the engineers developing the PhysioMio device may have influenced how participants perceived the interviewer’s stance. It is possible that this association led participants to assume that the interviewer was interested in promoting technology-supported rehabilitation, which may have limited their willingness to voice criticism. However, this concern was not substantiated during the interviews, as interviewees voiced both positive and negative opinions freely.

The interview dynamic also varied notably between patient and therapist groups. As a medical student, the interviewer shared a professional language and epistemic frame with physical therapists, which fostered rapport and deeper engagement. Therapists often raised relevant themes with little prompting, making the interviews feel more collaborative and data-rich. In contrast, interviews with patients, especially those with limited experience of technology-supported rehabilitation, required more guidance. JB’s prior inexperience as an interviewer made this more challenging and may have affected the depth or direction of some of the conversations. These asymmetries may have led to variations in the richness and granularity of the data collected from different groups of participants. To mitigate this, JB was enrolled in a training course to learn interviewing methodologies, and interview transcripts were regularly checked by one of her supervisors (BMZ), an experienced qualitative researcher, who gave JB feedback on how to strengthen her skills as an interviewer.

## Results

Our findings are presented as follows: First, we examine how autonomy as a relational capacity is renegotiated in care relationships involving technology such as the PhysioMio device. Next, we explore how recording and sharing data on movement influences the relationship between patients and physical therapists, how it reshapes the focus during in-patient training sessions, and the way patients experience their rehabilitation. Finally, we consider how the roles physical therapists assume within the therapeutic process are reshaped when introducing technology. This includes taking on a holistic perspective and continually reassessing what meaningful progress looks like in relation to each patient’s personal context, values, and lived experience, rather than in generic clinical terms.

### Negotiating Autonomy Relationally

Interviews illustrated how patients (re)negotiated autonomy in care relationships involving technology such as PhysioMio. On one hand, the technology can support their desires for self-sufficiency and autonomy. However, participants also identified perceived limits of patient autonomy and discussed its dependence on existing trusting therapeutic relationships.

#### Regaining Independence

Many interviewed stroke patients described their sudden loss of independence as one of the most difficult aspects of recovery. Several spoke about how frustrating it was to have to rely on others for basic tasks, and how they were afraid that they were not getting enough rehabilitation: “I felt like I had not enough physical therapy, maybe once a week” (patient 8). This was perceived as problematic, since both patients and therapists were convinced that regular training was key to successful rehabilitation. Thus, patients perceived technologies that enable independent home training as a powerful means of regaining a sense of agency and control and welcomed any opportunity to be supported in their training efforts.

Many patients believed that, provided the device was easy to wear and use, it could encourage regular training. With minimal effort, they believed they could maintain consistency in their exercises, embedding therapy in their daily routine on their own terms.

Ultimately, this is a motivation for me to continue training at home. Because, you know, when you come home, the physical therapy here in the rehabilitation clinic is being ignored, and the old habits from being at home come back. That’s just normal, human nature, and [the device] could help [counteract] that. -Patient 6.

However, therapists remarked that the success of the device depended on individual patients’ motivation and personality, noting use “depends a lot on personal attitude” (therapist 6). Some patients echoed this concern, reflecting that they might struggle with self-directed routines and would find it hard to stay motivated without direct supervision from a therapist. For these patients, the open-ended nature of home training felt like more of a burden than an opportunity.

#### Distributing responsibility

Navigating this additional responsibility presented a recognized challenge.

How much responsibility can I transfer to the patient? And how much responsibility do I have to assume myself, because the patient can’t? Either because they don’t want to, or maybe their circumstances don’t allow it? -Therapist 2.

This assessment also includes determining the appropriate level of therapy. Interviewees questioned whether patients had the necessary skills or confidence to make these decisions independently and were concerned that they would either overtrain or undertrain.

The first thing that comes to mind is personal responsibility and the question of whether I want or can assume that, whatever. The second thing is, from my experience, the over- or underestimation of one’s capacities, meaning training too much and then sleeping for hours. As a patient, I cannot correctly assess this. -Patient 3.

Both patients and physical therapists believed that ongoing support from a professional would be required during home training. Relatedly, some interviewees - both patients and therapists - were concerned that placing too much emphasis on independence might marginalize patients who are less able to self-manage. For example, patients “who have no connection to the digital might struggle” (patient 1). The perceived issue was that new technology might risk favoring patients who are more independent or technologically literate over those who need additional support, or who might not be suited to this kind of therapy at all.

Finally, both therapists and patients emphasized that the overall responsibility of physical therapy should not be transferred to the device. One therapist, for instance, insisted on the importance of therapists retaining control over the device to stop it if it became harmful for the patient.

It shouldn’t happen that the device becomes independent and out of control. It could happen that the device says that the patient should do this or that because the algorithm is built that way. And I realize afterwards that their spasm got worse because they reinforced the spastic patterns. -Therapist 3.

Patients mirrored this view, saying that they relied on their therapist for guidance, reassurance, and trusted human oversight:

I would say both are needed. The therapist and the device. Because the device is still being developed and can still be faulty or whatever. And if the therapist with his knowledge and experience vouches for it, then we do it. -Patient 6.

This indicates that introducing technology requires ongoing collaboration and communication between patients, physical therapists, and developers, highlighting the importance of maintaining these relationships in technology-supported environments.

#### Trusting Therapeutic Relationships

Patients and therapists highlighted that trust in the technology was often shaped by the trust patients placed in their therapist, especially when introducing technology.

You would need a therapist that you’ve had a couple of sessions with already, someone you know, someone that knows you. (…) Then it is easier if someone recommends (such a device) to you -Patient 13.

Therapists noted that patients would often mirror their therapist’s attitudes. If a trusted therapist dismissed a tool, patients would likely reject it as well. The PhysioMio developers also recognized that:

We concluded that we have to integrate therapists into the business model as well, if we want to spread the distribution of the device. If it’s too local and (the demand) only comes from patients, it’s difficult. Ideally, therapists should be convinced and say, ‘look, this is a great device, you should use it, it can help you’. -Developers.

In addition to the therapist’s influence during the initial introduction of the device, patients consistently emphasized the importance of regular contact with their therapist throughout the interviews. Several patients explained that ongoing interaction was essential to maintaining motivation to train and to learn new exercises properly. Therapists agreed that continuous human involvement played a vital role in supporting patients throughout the rehabilitation process, reinforcing the importance of interpersonal connection alongside technical or therapeutic interventions. The developers also stated no intention of marketing the device as a replacement for therapists. Instead, they emphasized the importance of maintaining a human element in the process, namely, someone who could verify that the device accurately reflected the patient’s progress.

In sum, the post-stroke rehabilitation process can leave patients feeling extremely vulnerable. Restoring and maintaining autonomy is a vital part of rehabilitation, and technological tools can provide valuable opportunities for self-directed recovery. However, successfully integrating them depends on a strong therapist–patient relationship, clear communication, and mutual trust. A person’s sense of independence is shaped by a variety of emotional, cognitive, and contextual factors, and therapists play a pivotal role in evaluating these elements while carefully balancing empowerment with providing the right level of support.

### Navigating Data Within the Care Relationship

While technology like PhysioMio offered opportunities for enhanced monitoring and individualized feedback, its introduction also raised questions about how data is interpreted, shared and acted upon within the therapeutic relationship.

#### Recording Home Training

Collecting data on patients’ home training activities can provide valuable insights into their rehabilitation progress. Most interviewed stroke patients did not object to data sharing as long as the purpose of tracking was transparent and meaningfully contributed to their recovery: “If the therapist sees (the data) and can use it to determine what we need to work on, I would not have a problem with (movement tracking).” -Patient 5.

However, it was important to patients that therapists would continuously re-evaluate what information was necessary and that they were given a choice of what information would be shared and when.

(It is important) to integrate a good communication design that allows patients to say if they don’t want certain information to be transmitted, like time stamps. And to explain why certain information might be relevant for a therapist, but that it is never mandatory. As long as the patient has control over their data, I think we are safe. -Patient 11.

Patients and therapists recognized that tracking offered the potential to improve continuity of care and boost patient motivation: “Yes (the control through the therapist) definitely has an influence, because he would scold me if he saw that I didn’t do anything and that would be a good thing.” -Patient 7.

Although participants recognized that this supervisory role could encourage patients to adopt healthy behaviors, some therapists were concerned that this positioned them as authoritarian figures rather than supportive, collaborative partners in the rehabilitation process. They were wary that such a dynamic could undermine the therapeutic relationship by shifting it away from empathy and shared decision-making towards control and compliance.

At this point, a control situation arises. And this has nothing to do with intrinsic motivation. The patient doesn’t learn to do something for their health on their own behalf, but because they know they are being observed. (…) It would be an erroneous trend because therapists have a different role. They should enable patients, because therapies end at some point. -Therapist 1.

While in the minority, some patients themselves echoed this tension, voicing concerns about the potentially negative effects of being closely monitored. One patient described the therapist’s oversight as too invasive, while another admitted that fear of being judged might prevent them from attending appointments altogether.

These reflections highlight that, although monitoring can encourage engagement, it can also create pressure and discomfort, particularly when patients feel scrutinized rather than supported. However, therapists also put the risks into perspective, believing that potential tensions could mostly “be defused by talking to patients beforehand” (therapist 4) and explaining that these features were in the patients’ best interest: “The aim is not to monitor them to dominate them, but to support them.” -Therapist 3.

These insights highlight the ambivalence surrounding monitoring in rehabilitation. While some patients were motivated by the observation, others found it invasive. Therapists were also wary of being perceived as controlling rather than supportive. However, as long as data recording is clearly purpose-driven and transparently communicated, the patients participating in this study were generally open to it.

#### Interpreting Data

One of the key strengths promoted by PhysioMio is its ability to provide detailed insights into muscle recovery. However, one therapist was skeptical about this, saying that it was “different whether I sense something or a device. I need to know, I need to feel. Adhesive tissue or something, that’s dexterity. How can devices replace that?” (therapist 2). Other therapists challenged this perception and strongly advocated for using sensor technology in rehabilitation, arguing that sensors could detect subtle changes in patient performance far more accurately than even the most experienced therapist could. In numerous interviews, patients expressed similar beliefs, seeing sensor-based data as an opportunity to tailor therapy more precisely, enabling personalized training that adapts dynamically to the patient’s progress.

Furthermore, both therapists and patients saw the tracking everyday movements outside of therapy sessions as an opportunity to evaluate how well skills learned in therapy are applied in daily life.

To see how my hand reacts throughout the day during everyday activities. Because there’s a difference if I execute specific movements in therapy or if I do movements spontaneously during everyday activities like brushing my teeth or hair, getting dressed, and so on, so I think, for a limited amount of time, (everyday tracking) would be helpful -Patient 2.

However, such data-rich environments introduce new complexities. Patients and therapists expressed concerns that producing excessive information could be overwhelming and occupy too much time during in-person sessions. One therapist reflected that “you have to consider what to do with the large amount of data. Can you process it? Do I have the time to analyze it?” (therapist 2). A patient said that they believed the challenge was “not to get buried by our own data. And to evaluate it purposefully. Because that is the danger, that you can do something and then you produce more and more data, and only then you question what to do with it” (patient 10). These worries reflect the fear that intense focus on measurable data might come at the expense of other aspects of the therapeutic process.

In response to patients’ desire to limit the amount of data shared with therapists and focus only on the most relevant information, the PhysioMio developers decided to summarize the data recorded throughout the day. This approach provides valuable insight into everyday movement while avoiding full transparency of patients’ daily activities. However, during specific training exercises, the device would record the movement in detail to provide feedback on how to improve it.

While interviewees recognized the potential of sensor data to provide valuable insights and enable more precise, real-time adjustments to therapy, both patients and therapists emphasized that data alone was insufficient. They underlined the importance of continuously re-evaluating the usefulness of data to allow its meaningful integration into care. They also recognized the importance of a collaborative relationship between all parties involved in rehabilitation.

#### Experiencing Visual Feedback

Many patients stated that visualizing even minor improvements in their data would provide them with a clearer indication that their efforts were worthwhile: “I take pictures, videos of movements, to compare and look back on in two weeks. Otherwise, you might miss the minimal progress.” (patient 13). They imagined that seeing these changes reflected in numbers or graphs would strengthen their confidence, offering reassurance that they were moving in the right direction and fostering a motivated approach to rehabilitation.

At the same time, some patients thought that visualizing stagnation or decline could be discouraging. One patient found it unhelpful to see feedback on fluctuating efforts every day. Another patient said that they would develop expectations about the numbers they wanted to see and would feel frustrated if these numbers were not achieved.

Several patients also found reassurance in the possibility of comparing their own progress with anonymized data from others. They saw an opportunity to learn from others and look for opportunities to improve their own rehabilitation programs. Another explained that such comparisons could help them feel less alone and offered them an orientation in their recovery process. Yet, other patients were concerned that comparisons would be unfair and useless, given that recovery trajectories are rarely linear and vary greatly from one person to another.

It is problematic, because you are talking about individuals with different lives, circumstances, characters. Some might be motivated by what someone else achieved; someone else would not (…) Everyone has a different starting point. -Patient 3.

Therapists also stressed the complex and inherently personal nature of rehabilitation processes. They emphasized that single data points should not be given too much weight when assessing the overall rehabilitation process.

We still know little about why certain functions don’t come back, even though, based on theoretical prognosis, they should. Or sometimes functions spontaneously come back and we cannot figure out why they do for some patients and not for others. -Therapist 1.

These findings illustrate that while patients welcomed the possibility of visible progress, they also voiced concerns about how confronting perceived setbacks might affect their motivation and emotional state. Developers reacted to these findings by adapting the feedback display: While they initially had planned comparisons with other patients’ data, they decided instead to compare the patient only to their personal starting point.

### Maintaining a Holistic View on Care

The interviewees also reflected how roles and responsibilities between patients and physical therapists would be reshaped by technology. They reflected on the relational foundation necessary to ensure that rehabilitation feels meaningful and relevant to individual patients.

#### Therapists’ Role

When talking about technology-supported rehabilitation, therapists reaffirmed their role in maintaining a holistic and situated perspective on care. They mentioned their responsibility to consider not only muscle activity, but also psychological states, personal goals, and social circumstances.

Especially in the beginning, a big aspect is providing psychological support. You naturally take care of physical ailments, (…) but the most important therapeutic step is to give the patient a sense of hope. -Therapist 4.

This holistic approach to care was not only how therapists saw their role, but also how patients perceived them. Therapy was valued not only for its physical benefits but also for the emotional and psychological support it provided. Sometimes “the most important part is not the content of the rehabilitation itself, but the relationship between the patient and therapist” (therapist 1), with therapists seen as important sources of comfort and knowledge.

Beyond the psychological dimension, patients emphasized the importance of viewing the whole body during recovery. Although they recognized that technology could provide detailed information about specific muscle activity, they believed that therapists were crucial for understanding the bigger picture, including posture, alignment and other factors that devices could not fully capture.

The device obviously is more precise. Because it registers every detail. But the physical therapist has a more, let’s say, holistic approach. The device looks at the muscles, or its measurements. But not at the spine, or posture, or other factors that influence my movements. I therefore think it is crucial that you look at the human as a whole. -Patient 8.

This perspective put the fear of technology replacing physical therapists into perspective. The relational care provided by therapists meant that participants could not “imagine a human being replaced in all their complexity” (therapist 5). Instead of fear, interviewees focused on how “modern technologies should be seen as support and assistance rather than a replacement for humans” (patient 2). Additionally, “the data from such devices could open up new areas of research” (therapist 4), creating new opportunities to grow.

#### Space Beyond Therapy

When discussing the importance of contextual, holistic care, another important issue was the need to establish clear temporal and spatial boundaries for care. Therapists stressed the importance for patients to step outside the role of the care recipient and protect spaces where their identity was not defined by illness or rehabilitation. In their experience, when patients were constantly in ‘recovery mode’, even outside of formal therapy sessions, it led to psychological fatigue and reduced motivation. Fewer, but more conscious training efforts were more valuable than constant training.

Several interviewees stressed the importance of using the device only during designated training periods. Although everyday tracking is potentially valuable for data collection, it was seen as an intrusion into private life. Examples of clearly defined boundaries included choosing not to wear the device during holidays or at work: “I would not want to wear (the device) all day long. But maybe for an hour or if I’m doing something where it does not bother me” (patient 9).

These views were revealing for the PhysioMio developers, who had not anticipated that more data collection was not always better from the perspectives of patients and therapists:

We had the vision that patients would wear the device all day, because the generated data would be interesting. That is a developer’s point of view: all data is relevant. And then […] we realized: people don’t want to wear the device all day. -Developers.

Patients’ experiences of recovery revealed that rehabilitation is not a uniform process and that physical improvement is not always the primary goal. Some patients wanted to focus on their cognitive abilities, others prioritized a quick return in their home environment, even if that was at a certain detriment to their recovery. Therapists were expected to recognize and adapt to these individual priorities, continually reassessing what meaningful progress looked like in relation to each patient’s personal context, values, and lived experience, rather than in generic clinical terms.

In sum, throughout the interviews, the use of data in rehabilitation was considered valuable but complex. While recording progress could motivate patients, it also raised concerns about surveillance and control within the therapeutic relationship. Participants emphasized that data must be interpreted within the broader context of the patient, accounting for daily fluctuations, emotional state, and individual goals. Rather than relying on numbers alone, patients and physical therapists alike stressed the need for a holistic approach where technology supports, rather than replaces, human insight and connection.

These accounts show that rehabilitation technologies are not neutral tools. They actively influence how recovery is experienced, negotiated, and integrated into daily life. They create new relational possibilities and challenges, which will be reflected on through an ethics of care lens in the following discussion.

## Discussion

By analyzing qualitative interviews with stroke patients and physical therapists about the sensor-based rehabilitation device PhysioMio, this study illustrates how technology-supported rehabilitation reshapes care relationships between patients and physical therapists. Its findings complement studies conducted in other care settings that highlight the importance of therapeutic relationships in providing technology-assisted care. In a scoping review on a care ethics framework for midwifery practice, Buchanan et al. (2022) discussed the positive effect of strong therapeutic relationships on ethical sensitivity. A study from a geriatric hospital in Finland by Juujärvi et al. (2019) argued that building relationships with their patients made nurses better aware of their needs and personalities, therefore enhancing their ethical awareness. Similarly, in a study with nurses working in a British general hospital, Barlow et al. (2018) identified nurses’ relationships with patients as an important factor for achieving the best outcome for patients during ethical dilemmas. Our paper contributes to care ethics scholarship by examining how the introduction of technology reconfigures the relational dynamics of rehabilitation care in concrete and nuanced ways.

Our findings illustrate how introducing technology into the care process changes the two-agent dynamic to a three-agent dynamic, including the technology as an additional agent. The following sections each focus on one dimension of the three-agent dynamic: (1) the shift in patients’ role within the therapeutic relationship; (2) how therapists navigate providing care in technology-supported contexts; and (3) how patients engage with and establish trust in the technology as a new relational agent in their care.

### Patients’ Evolving Role Within Technology-supported Therapeutic Relationships

Our findings illustrate that the technology offers patients an increased capacity of participation in the therapeutic process. For instance, PhysioMio offers additional training opportunities outside of therapy sessions. Although the effects of PhysioMio on training habits cannot be definitively predicted at this early stage of development, a positive impact on long-term therapy adherence is likely, given the results of other studies. For example, Souza et al. (2025) found a strong correlation between patients’ perceived self-efficacy and long-term adherence to therapy in musculoskeletal rehabilitation.

At the same time, interviewees noted that assuming a more active role in their rehabilitation can be daunting, for example, when determining training intensity. When this decision is shifted from the therapist to the patient, part of “the burden of treatment” is also shifted (Lupton, 2013, p.8). It should be noted here that this might be particularly challenging for patients at the beginning of their stroke rehabilitation, who often have little previous experience with physical therapy and may, therefore, be particularly vulnerable. Ethical care therefore must acknowledge individual differences in ability, motivation, and technological affinity and adapt to them (Ienca et al., 2021; Spink et al., 2021).

Further, our findings illustrate that increased opportunities for patients to shape their rehabilitation do not lead to a detachment from therapists. On the contrary, as a study by Fiske et al. (2020) analyzing different medical practitioners’ experience with digital self-care also supported, personal relationships are essential for safe and effective use of digital health applications. This reflects the care ethics understanding that autonomy does not mean complete independence from others, but should rather be understood as a capacity embedded in and nourished by strong, trusting relationships with care providers (Delgado, 2019).

Another possible shift in the patient-therapist relationship emerged during the discussion of therapists’ ability to monitor patients’ home training via collected data. A conflict may arise when introducing devices with such data collection features, as patients might change their behaviors because they are being monitored, not because they are intrinsically motivated (Lucivero & Jongsma, 2018). While our findings support the potential for such a tension, they also suggest that this tension can be mediated by establishing collaborative, trusting relationships between patients and healthcare providers before introducing medical technologies such as PhysioMio. Physical therapy may be a particularly promising context for introducing such technologies, as the long-term nature of rehabilitation provides opportunities to build the trust and relational foundation necessary for navigating these tensions.

### The Influence of Technology on Therapists’ Capacity to Provide Relational Care

Another relevant perspective emerging from this study is how technology influences the care provided by therapists. From an ethics of care perspective, such focus on patients’ individual needs improves care (De Panfilis et al., 2019). Andersson et al. (2017) found that caregivers in an elderly care facility felt that technological devices compromised their ability to focus on patients as unique persons by distracting them from being present in the moment. This could compromise the relationship and communication between patient and therapist. Our study gives further nuance to this aspect: Although stroke patients and physical therapists expressed concerns that overly granular information could be overwhelming and distract from the bigger picture, they also recognized the potential of technology like PhysioMio to support attentive, personalized care. The sensor’s ability to quickly and accurately detect subtle changes in patient performance provides opportunities to adjust difficulty levels and offer more tailored rehabilitation exercises in real time. The challenge lies in balancing the benefits of increased data with the distractions it creates.

Participants further highlighted the importance of therapists as interpreters of context, data, and individual needs. This finding of our study is mirrored in other studies. For example, it reflects medical doctors’ assessment that they have a responsibility to “contextualize results within the broader picture of [patients’] health and life goals” (Fiske et al., 2020, p.8). It is also reflected in a study by Mort et al. (2003) on telemedicine in dermatology, who concluded that reliable judgment not only depends on images, but requires “a patchwork of other kinds of activities and materials, such as reassurance, explanation, history taking, intuitive investigation” (p. 12). As in these other healthcare domains, our study underlines that while additional data can be beneficial for stroke rehabilitation, patients should not be reduced to mere data points but must be understood in their full emotional, relational, and contextual complexity. The ability to adopt such a holistic view sets human caregivers apart from technology.

This insight also puts concerns about therapists being replaced by machines into perspective. One aspect of this concern is voiced by Jongsma et al. (2021), who discuss the risk of health care professionals losing their “trained gut feeling” (p. 5) because they limit their focus on numbers and data. By foregrounding the relational aspects of care, our findings support Osuji’s (2018) argument that “the labor of care cannot, for the most part, be replaced by machines” (p. 3). While technology can support some aspects of care, such as precise movement measurements, therapists are essential to maintain the relational aspects of care (Mesko & Győrffy, 2019): trusting therapists, supporting patients’ self-efficacy without leaving them unsupported, and navigating patients’ interactions with the device regarding self-image or privacy. However, it is important to note that hands-on movement measurements can be an important aspect of a physical therapist’s identity. A shift from direct physical observation to sensor-based measurement therefore requires sensitive handling, as it reconfigures a core aspect of how physical therapists relate to and understand their patients. Consequently, discussing technology in physical therapy settings requires particular sensitivity when anticipating and supporting the necessary changes in skill sets to reframe the potential “deskilling” or replacement of therapists’ skills as an opportunity for them to develop new skills and take on new roles (Chua & Kuah, 2017).

### Patients’ Relational Engagement with Technology

Our findings illustrate that patients do not engage with the device in a simply neutral way: How feedback is presented triggers an emotional response in many patients, some feeling motivated, others judged. Mediating health care via technology therefore not only influences the relationships between users and healthcare providers, but creates an emotional relation to the device itself (Lupton, 2013). It is essential to recognize these dynamics and consider how patients interact with the technology. Some important aspects are consent and privacy. Contrary to our expectations, many patients expressed no objection to the device recording movement data to share it with their therapist, provided it was purpose-driven and meaningfully contributed to their rehabilitation. Even though this finding is consistent with Wang et al. (2023), who found that “issues of privacy loss may be mediated by perceptions of the smart home technology’s usefulness” (p. 4) and Ienca et al. (2021), who in a study on health interventions for elderly patients found that “participants appeared generally willing to sacrifice a portion of their privacy as a trade-off to the benefit of safety and independent living” (p. 8), this finding is noteworthy because of the German study context: In Germany, data privacy – especially in medical contexts – is highly valued and saliently discussed. Robust data protection legislation (such as the European General Data Protection Regulation and the Federal Data Protection Act) and widespread public awareness of privacy issues illustrate high sensitivity of handling personal data.

Many patients considered privacy not an absolute value in isolation, but rather as a relative value that can be negotiated within a trusted care relationship. Our findings illustrate that one part of that negotiation was patients’ desire to decide what data was shared and when. Thus, developers of health technologies should implement flexible data sharing options. From an ethics of care perspective, informed consent is not a one-time formality or merely a matter of legal compliance, but an ongoing, relational practice grounded in mutual understanding (Sharkey & Sharkey, 2012; Tannou et al., 2020). Even though asking for patient consent once at the beginning of using PhysioMio is the legally required procedure, it is insufficient from an ethics of care perspective. Instead, the usefulness of data collection through the device should be continuously re-evaluated throughout the rehabilitation process. Patients should have the option to adjust which information is shared (Lucivero & Jongsma, 2018). Wang et al. (2023) emphasized further that consent is only possible if users understand the technology, its purposes and uses. From an ethics of care perspective, ethical use of the PhysioMio device is therefore only possible if the system is transparent, accessible and intelligible for its users.

### Strengths and Limitations

While the study provides nuanced insights into how the use of technology-assisted rehabilitation affects care relationships between patients and therapists, it also has limitations. First, the small number of participants, especially physical therapists (*n* = 7) due to limited resources, time constraints, and recruitment challenges, restricts the generalizability of the findings. Nevertheless, data collection continued until no substantial new insights emerged and the interviews were conducted in considerable depth and yielded detailed and varied accounts. The combination of different viewpoints – stroke patients, physical therapists, and the PhysioMio developers – allows this study to represent a variety of perspectives on how care relationships are affected by technological devices such as PhysioMio.

Second, the study was conducted with participants living in Bavaria, Germany. This region represents a high-resource setting where everybody has access to public or private health insurance. Yet, this also means that access to new technologies depends on whether they are included in the reimbursement lists of health insurances – otherwise, clinical centers and physical therapists may not adopt them. Other healthcare systems might shape patient and provider perceptions and their care relationships differently and are not represented in this study.

Third, this study operated on a concrete case, the PhysioMio device. While case studies allow for applied, concrete discussions with interview participants, the findings mostly apply to health technologies used in similar contexts. Nevertheless, findings from other studies reveal that similar issues related to data use and care relationships are discussed in other settings, indicating that the findings bear broader relevance.

Finally, the interviews were introduced with a description of the device and its intended use. The wording and framing of this description may have influenced participants’ responses. For example, the idea of patients using the device independently from a therapist was introduced by the interviewer, as this was an essential feature of the imagined device. While the authors acknowledge that such framing may have influenced the language participants used, the substantive ideas that emerged, such as concerns about home use, handling technical difficulties, or determining training intensity, were raised organically by participants. The interview questions also asked specifically about the patient-therapist relationships and how they might be influenced by technology. This means that the concept was not explored instinctively by participants. However, as the patient-therapist relationship was the explicit focus of this study, directing participants’ attention to this topic was a deliberate and methodologically justified decision.

### Conclusion

This study illustrates that the ethical use of medical technology, such as PhysioMio, must be grounded in trusting therapeutic relationships and adapted to patient-specific circumstances. When attuned to individual needs and abilities, and when thoughtfully developed and integrated, health technologies like PhysioMio can empower patients, enhance therapists’ roles, foster trust through purposeful data use, and provide motivational feedback without overwhelming either party. However, when deployed without such consideration, they risk undermining self-efficacy, eroding trust, and distorting relational dynamics. In addition to medical effectiveness and safety assessments, the responsible implementation of medical technologies is crucial.

## Data Availability

As it is not possible to fully anonymize interview transcripts, and as we do not have the participants' consent to publish them, we are legally unable to make the full transcripts available. However, data excerpts may be available from the corresponding author upon reasonable request.
